# Unethical Advertisements

**Published:** 1878-03

**Authors:** 


### Editorial.

### UNETHICAL ADVERTISEMENTS.

The founders of our present medical code took a proper view of human nature when they condemned the lauding of medical men for extraordinary cures, by hand-bills, circulars, or advertisements in secular papers. The afflicted are favorably impressed by ingeniously-gotten-up reports of cures, thrust upon them in every conceivable way, and hence the most liberal and unscrupulous advertiser is consulted, though he may be entirely destitute of medical and surgical qualifications. The professional prohibition, intended more for the protection of the public against those who seek fame without medical knowledge, than otherwise, signally fails of its object by not having any control of those outside the profession. The distinction is not known to the public, and if the prefix "Dr." or appendage "M.D." accompanies the advertiser's name, he is supposed to be entitled to their confidence. Men who know as little of medicine as those they seek to dupe, assume the title with impunity, and thus the world moves on.

We are frequently interrogated in regard to the qualifications of outsiders in the city, advertising specialties, and the answer we give is doubtless often attributed to jealousy toward the celebrated advertiser. Barnum understood human nature when he said mankind wants and must have humbuggery. It is, therefore, probably the best answer for such enquirers to say, go and be humbugged, and then you will know for yourself. Honorable, intelligent physicians throughout the country are often deceived by such specialists, and send their patients to them, supposing they are regularly connected with the profession.

As we say to them privately we now say to all—whenever, in circulars or newspaper advertisements, extraordinary cures, with or without pictures accompanying them, are paraded before the public, the writer is seeking to make known advantages over other practitioners which he does not possess, and is likely an unmitigated fraud.

